# The Lysosome in Malignant Melanoma: Biology, Function and Therapeutic Applications

**DOI:** 10.3390/cells11091492

**Published:** 2022-04-29

**Authors:** Chia-Hsin Hsu, Keng-Jung Lee, Yi-Han Chiu, Kuo-Ching Huang, Guo-Shou Wang, Lei-Po Chen, Kuang-Wen Liao, Chen-Si Lin

**Affiliations:** 1 Department of Veterinary Medicine, School of Veterinary Medicine, National Taiwan University, Taipei 10617, Taiwan; ch932@cornell.edu; 2 Department of Biomedical Sciences, Cornell University, Ithaca, NY 14853, USA; 3 Department of Biomedical Engineering, Carnegie Mellon University, Pittsburgh, PA 15213, USA; kengjunglee@cmu.edu; 4 Department of Microbiology, Soochow University, Taipei 10617, Taiwan; chiuyiham@scu.edu.tw; 5 Holistic Education Center, Mackay Medical College, New Taipei City 25245, Taiwan; kchsports@mmc.edu.tw; 6 Department of Biological Science and Technology, National Yang Ming Chiao Tung University, Hsinchu 30068, Taiwan; b8905043@gmail.com (G.-S.W.); liaonms@g2.nctu.edu.tw (K.-W.L.); 7 Ph.D. Degree Program of Biomedical Science and Engineering, National Yang Ming Chiao Tung University, Hsinchu 30068, Taiwan; robert82ccc@gmail.com

**Keywords:** lysosome biogenesis, chemoresistance, melanoma therapy target

## Abstract

Lysosomes are membrane-bound vesicles that play roles in the degradation and recycling of cellular waste and homeostasis maintenance within cells. False alterations of lysosomal functions can lead to broad detrimental effects and cause various diseases, including cancers. Cancer cells that are rapidly proliferative and invasive are highly dependent on effective lysosomal function. Malignant melanoma is the most lethal form of skin cancer, with high metastasis characteristics, drug resistance, and aggressiveness. It is critical to understand the role of lysosomes in melanoma pathogenesis in order to improve the outcomes of melanoma patients. In this mini-review, we compile our current knowledge of lysosomes’ role in tumorigenesis, progression, therapy resistance, and the current treatment strategies related to lysosomes in melanoma.

## 1. Introduction

Lysosomes are membrane-enclosed organelles responsible for the disposal and recycling of worn out and damaged cellular macromolecules and organelles. Cells digest foreign materials by endocytosis and phagocytosis in lysosomes [1,2]. Lysosomal degradation products are recycled back to the cytosol via diffusion and specific transport channels or released to the extracellular space by exocytosis [1]. Moreover, lysosomes play essential roles in other cellular processes, including nutrient sensing and the control of energy metabolism [3].

The alteration of lysosomal functions can lead to broad detrimental effects, such as inflammation, apoptosis, failure to clear potentially toxic cellular waste, and the dysregulation of cellular signaling, causing a variety of diseases, including cancers [3]. It is expected that the expression and function of various lysosomal hydrolases are increased in human tumors, and they often correlate with a higher risk of recurrence and poor prognosis [1]. In addition, studies have shown that lysosomal enzymes play critical roles in cancer invasiveness, angiogenesis, and progression [1].

Malignant melanoma, a highly metastatic, drug-resistant, and aggressive malignancy, is the most lethal form among skin cancers [4]. Although it comprises only 5–10% of all skin cancers, malignant melanoma is responsible for more than 75% of all skin cancer-related deaths [4]. The incidence rates of melanoma have been augmenting rapidly over the past few decades. According to American Cancer Society’s estimates, about 99,780 (57,180 in men and 42,600 in women) new melanomas will be diagnosed in the United States in 2022 [5]. Several risk factors are reported to be associated with cutaneous melanoma, including skin type (fair skin, freckling, and light hair), exposure to sun radiation, number of nevi (>50 moles), age (being older), gender (being male), immune status (having a weakened immune system), family history and former removed melanomas [6]. The early stages of melanoma are relatively easy to manage by surgical excision. However, melanoma is highly invasive and metastatic, and the prognosis for long-term survival is poor when the disease reaches advanced stages. Even though the use of targeted therapies and immunotherapies has improved the clinical outcomes, melanoma remains notoriously difficult to treat once it metastasizes to other sites, including brain, lungs, liver or bone [7].

Increasing efforts are currently focusing on novel biomarker identification for predicting the treatment response and prognosis of melanoma. Zhang et al. established a 14 cytolytic activity (CYT)-related-gene prognostic model, which can be used as a predictive biomarker and therapeutic target for cutaneous melanoma patients [8]. Bacchetti et al. reported that the expression levels of paraoxonase-2 (PON2) could be positively related to the tumor aggressiveness of skin cancers, including melanoma [9]. Campagna et al. demonstrated that nicotinamide N-methyltransferase (NNMT) knockdown led to a significant reduction in cell proliferation and migration of human melanoma cell lines and was correlated to enhanced chemosensitivity to dacarbazine, suggesting that NNMT could represent a molecular target for the effective treatment of melanoma [10]. Still, it is important to understand melanoma pathogenesis and factors that contribute to drug resistance to develop new therapeutic strategies.

In this review, we mainly focus on the role of lysosomes in melanoma tumorigenesis and progression, along with therapy resistance, including chemotherapies, targeted therapies, and immunotherapies. We also highlight the current treatment strategies related to lysosomes in melanoma.

## 2. The Roles of Lysosomes in Melanoma Progression

Hanahan and Weinberg proposed that cancers have several hallmarks, such as sustaining proliferative signaling, evading tumor suppressors, and promoting invasion and metastasis [11]. Lysosomes play critical roles in tumorigenesis and cancer progression. Rapidly proliferating cancer cells place heavy demands on the synthesis rates of new proteins, membrane lipids, DNA, and RNA [12]. In addition, cancer cells need to compete with extreme fluctuations in the poor vascularized tumor microenvironment with limited nutrient and oxygen levels [13]. Thus, nutrient-scavenging pathways associated with lysosomes help generate the nutrients and energy required by cancer cells [13]. Compared to healthy tissue, melanoma has higher level of reactive oxidative species (ROS), which can damage DNA, proteins and lipids, resulting in senescence and cytotoxicity [14,15]. Lysosomal autophagy helps melanoma cells escape senescence and promote adaptive survival by recycling damaged cellular organelles and proteins [16].

### 2.1. Lysosomes Mediate Melanoma Cell Proliferation, Survival and Death

In mammalian and yeast cells, the master growth regulator mTORC1 protein kinase is recruited and activated at the lysosome in response to nutrients [17]. In tumor development, cancer cells often experience stressed and nutrient-limited milieu. Through phagocytosis, endocytosis, and macropinocytosis, extracellular substances are delivered to lysosomes and undergo lysosomal degradation to generate nutrients [18]. In melanoma, it is reported that Ca^2+^ channel mucolipin 1 (MCOLN1) is preferentially required for the survival and proliferation of melanoma cells by negatively regulating MAPK and mTORC1 signaling [19]. Additionally, macropinocytosis is upregulated in melanoma cells relative to normal melanocytes and is sustained by MCOLN1 [19].

The MiT/TFE transcription factor family, encoding MITF (micropthalmia-associated transcription factor), TFEB, TFE3, and TFEC, belongs to the MYC superfamily of basic helix-loop-helix leucine zipper (bHLH-ZIP) proteins [20]. MITF is recognized as a master regulator of melanocytes required for the development, growth and survival of melanocytes and as a melanoma oncogene amplified in 30–40% of melanomas [21,22,23]. Additionally, it is observed that the MITF E318K mutation predisposes to familial and sporadic melanoma [24]. MITF in melanoma not only acts as a major oncogene, but also correlates with many lysosomal genes and generates late endosomes that are not functional in proteolysis [25]. MITF is also the transcriptional regulator of v-ATPase gene expression in melanocytes and melanomas [26,27].

Ploper et al. reported that, in melanoma cells, the nuclear accumulation and stabilization of MITF caused an expansion of the late endolysosome/multivesicular body (MVB) compartment and elevated expression of late endosomal proteins, including Rab7, LAMP1, and CD63. This increased endosome/MVB biogenesis enhanced Wnt signaling by increasing the sequestration of destruction complex components, such as GSK3 and Axin1 inside MVBs, generating a positive feedback loop and ultimately contributing to melanoma proliferation [22,25]. Based on this finding and the fact that MITF-M isoform, which is the type of isoform expressed in melanomas, lacks this N-terminal domain which is required for lysosomal localization and mTOR phosphorylation, it is proposed that overexpressing MITF-M binds promiscuously to CLEAR element lysosomal genes without being restrained by mTOR signaling and hence regulates endolysosomal biogenesis [25].

Similarly, Möller et al. demonstrated that MITF can bind to the CLEAR-box element in the promoters of lysosomal and autophagosomal genes in melanocytes and melanoma cells [28]. In metastatic melanoma cell lines and tumors, MITF positively correlated with the expression of lysosomal and autophagosomal genes, and the knockdown of MITF resulted in starvation-induced autophagy degradation in both melanocytes and melanoma cells, presumably due to less autophagosomal formation [28]. On the other hand, the overexpression of MITF in melanoma cells elevated the expression of lysosomal and autophagosomal genes and induced autophagosome formation, but not sufficiently to induce autophagic flux [28], echoing the finding observed by Ploper et al. [25].

Furthermore, it is reported that an MITF-dependent melanoma patient-derived cell line, 501Mel, showed induction of RagD expression and increased mTORC1 activation, and the silencing of RagD was sufficient to greatly revert the hyperproliferative phenotype of this melanoma cell line [29]. Notably, markedly reduced xenograft tumor growth upon RagD silencing was observed in mice bearing 501Mel melanoma cells, indicating a key role of RagD in promoting tumor growth [29]. A significant correlation between MITF and RagD gene expression levels is demonstrated by the analysis of microarray data of melanoma metastatic patients and melanoma cell lines [29]. These findings support the idea that melanomas associated with MITF hyperactivation lead to constitutive RagD GTPase transcriptional induction and enhanced mTORC1 signaling, which fuels tumor growth [29].

On the other hand, melanoma cells with enhanced expression of LAMP-2C, a lysosome-associated membrane protein, displayed elevated cell cycle arrest, increased the expression of the cell cycle regulators Chk1 and p21, and greater apoptosis and necrosis [30]. Reduced tumor growth was also observed in immune-compromised mice bearing melanoma cells with increased LAMP-2C expression, suggesting a potential role for LAMP-2C as a tumor suppressor in melanoma progression [30].

### 2.2. Lysosome Alterations Promote Melanoma Invasiveness and Metastasis

Cysteine cathepsin proteases (herein referred to as cathepsins) are involved in almost all processes related to lysosomes, such as protein degradation, protein and lipid metabolism, autophagy, antigen presentation, growth factor receptor recycling, cellular stress signaling, and lysosome-mediated cell death [31]. In addition, these enzymes can be secreted via lysosomal exocytosis, resulting in the degradation of extracellular targets [31]. Accumulating evidence suggests that cathepsins critically contribute to tumor progression in a variety of cancers [31]. Cathepsins are crucial acid hydrolases within lysosomes and are the key effectors of protein catabolism and autophagy, which support the elevating metabolic needs of proliferating cancer cells [31,32,33]. Moreover, secreted cathepsins can modify the tumor microenvironment by degrading the extracellular matrix and contribute to tissue invasion and metastasis [31,34,35,36].

In invasive human melanomas, cathepsins B and L were found to be upregulated and were correlated with metastasis. The inhibition of the cell-membrane-permeable cathepsins B and L suppressed the invasive growth of the melanoma cells [37]. Similarly, cathepsin K was strongly expressed in most primary melanomas and all cutaneous melanoma metastases. The inhibition of cathepsin K significantly reduced melanoma cell invasion and increased detection of internalized collagen in vitro [38]. Additionally, Alonso-Curbelo et al. reported that cathepsins can be misrouted within the cell via the lineage-specific wiring of the endolysosomal pathway [39].

Rab7, a GTPase, has a key role in lysosome biogenesis and the lysosomal-associated degradation of cytoplasmic vesicles. The knockdown of Rab7 promoted the secretion of multiple lysosomal cathepsins and matrix proteins, and subsequently increased melanoma invasion [39]. Moreover, Tripathi et al. investigated the signaling pathways leading to increased cathepsin secretion in melanoma cells and found that the nonreceptor tyrosine kinases Abl and Arg induced the secretion of cathepsins B and L by activating transcription factors, including Ets1, Sp1, and NF-*κ*B/p65, which play critical roles in driving melanoma invasion and metastasis [40].

Furthermore, Johansson et al. revealed that the oncogene PRL3 bound and dephosphorylated RNA helicase DDX21 to restrain the transcription of MITF-regulated endolysosomal genes in the melanocyte stem cell [41]. In clinical melanoma samples, PRL3 expression was inversely correlated with endolysosomal vesicle gene expression, and high PRL3 expression was significantly associated with melanoma-specific death, making it a valuable predictive marker for metastatic melanoma-specific death in all stages [41]. Together, the abovementioned studies support the idea that the dysregulation of endolysosomal pathways has emerged as a hallmark of melanoma and a driver of metastasis (Table 1) [39,41,42].

## 3. Lysosomes and Therapy Resistance

### 3.1. Lysosomes Mediate Chemoresistance

Lysosomes play important roles in resistance to therapies such as chemotherapy, targeted therapy, and immunotherapy. Prior to recent therapeutic advances, chemotherapy was the treatment of choice for advanced melanoma, although the success has been minimal due to chemoresistance [44]. It may, however, remain important in the palliative treatment of refractory, progressed, and relapsed melanoma [45]. The molecular basis of chemoresistance in melanoma is multifactorial, including the overexpression of drug efflux proteins, the alteration of enzyme activation, the deregulation of apoptosis, Ras mutation, epithelial to mesenchymal transition, and the deregulation of microRNA expression [46].

Lysosomes contribute to hydrophobic weak base chemotherapeutic drug resistance via lysosomal sequestration, based on passive cation trapping [47]. Hydrophobic weak base chemotherapeutics become entrapped in lysosomes due to their protonation in the acidic lumen of lysosomes. Hence, these sequestered drugs can neither reach their target sites nor exert their cytotoxic effects [47]. In addition to the passive accumulation of chemotherapeutics, lysosomes can also mediate active lysosomal drug sequestration via transporters localized in the lysosomal membrane [47].

Autophagy is also emerging as a key player in therapy resistance. Therapy-induced autophagy has two possible effects; it may either contribute to the anticancer effects of chemotherapeutics or facilitate chemotherapy resistance [48]. In melanoma, Ma et al. showed that patients bearing melanomas with a high autophagic index were less likely to respond to treatment (temozolomide and sorafenib), and autophagy inhibition using either hydroxychloroquine or inducible shRNA against Atg5 led to a significant augmentation of temozolomide-induced cell death [49]. On the other hand, Li et al. demonstrated that the blockade of BRAF inhibitor-induced autophagy–lysosome activation in melanoma xenografts results in chemoresistance, which is associated with elevated TGF-β levels and enhanced TGF-β signaling [50].

### 3.2. Lysosomes Promote Resistance to Targeted Therapies

Kinase inhibitors, particularly BRAF and MEK inhibitors, have become the first line of treatment for advanced BRAF-mutated melanoma, which is the most common mutation in cutaneous melanoma. Although the response of these targeted therapies is impressive, the duration is short lived, with acquired resistance after a median of 6–8 months [51]. It is known that therapy-induced autophagy promotes resistance to kinase inhibitors. Ma et al. investigated tumor biopsies from patients treated with BRAF inhibitors or combined BRAF/MEF inhibitors and found that tumors with resistance to BRAF inhibitors had increased levels of autophagy relative to the baseline [52]. In addition, patients with higher therapy-induced autophagy levels experienced significantly lower response rates to BRAF inhibitors [52]. The in vitro experiments further demonstrated that BRAF inhibitors induced cytoprotective autophagy by activating an ER stress response [52]. Interestingly, it is shown that heterozygous (but not homozygous) Atg5 loss compromised the response of BRAF inhibitors in melanoma-specific mouse models, raising caution about the incomplete blockade of this gene in clinical aspects, since this may cause an unexpected worsening of patient outcomes [53].

### 3.3. Lysosomes Contribute to Immunotherapy Resistance

Immunotherapies with CD8+ cytotoxic T cells as major cellular effectors appear to be a promising treatment option for patients with advanced melanoma, since they show durable response rates in selected patients [7]. However, primary and acquired resistance to immunotherapies is common [7]. The secretion of the pore-forming protein perforin and granzyme B by human cytotoxic T cells is the crucial mechanism to kill target cells, which occurs at the cytotoxic T cell/target cell lytic synapse [54,55]. It is reported that human melanoma cells can develop a rapid response to cytotoxic T cells at the lytic synapse by a secretory burst of lysosome/late endosomes, which leads to cathepsin-mediated perforin degradation and deficient granzyme B penetration [55]. The inhibition of this melanoma response by the depletion of SNAP-23-dependent lysosomal trafficking, pH perturbation, or impairment of lysosomal proteolytic activity restores susceptibility to cytotoxic T cell assault. This finding supports this potential mechanism contributing to melanoma cell immune resistance [55]. Moreover, dendritic cells are required to license the cytotoxic activity of CD8+ T cells in T-cell-based immunotherapy. Santana-Magal et al. found that tumor-infiltrating monocyte-derived dendritic cells undergo apoptosis due to their excessive phagocytosis of melanoma-secreted lysosomes as melanoma progresses. In their absence, cytotoxic T cells fail to lyse melanoma cells, suggesting that lysosomes play a role in limiting the effect of immunotherapy [56].

## 4. Targeting Lysosomes in Cancer Therapeutics

The contribution of lysosomes to cancer progression and therapy resistance provides the rationale for targeting lysosomes as a cancer therapeutic strategy. Lysosomes are critical for both anabolic growth pathways driven by mTORC1 and catabolic pathways, including macropinocytosis as well as autophagy; these pathways are all potential targets in cancer [57]. mTOR is crucial for regulating cell growth, proliferation, metabolism, and angiogenesis. mTOR inhibitors have been widely used against cancers in multiple preclinical studies and clinical trials [57,58]. Despite having both pro- and antitumor effects, autophagy is a potential target of inhibition because it can promote the growth of established tumors and mediate therapy resistance during cancer therapy [59].

### 4.1. Lysosomotropic Agents

Lysosome-targeting strategies mainly focus on lysosomotropic compounds, such as chloroquine, ammonium chloride, methylamine, and siramesine [60]. Because of their weak base characteristics, lysosomotropic compounds act by elevating lysosomal pH when accumulating in the lysosomal lumen and usually inducing lysosomal membrane permeabilization [60,61]. Additionally, as repurposing approved and abandoned nononcological drugs becomes an alternative approach to identifying anticancer therapeutics, antimalarials that potentially inhibit lysosomal functions have been considered as promising candidates for oncological repurposing and have been tested in preclinical and clinical cancer studies [62,63]. Hydroxychloroquine (HCQ), one such antimalarial, has been widely investigated in many clinical trials combining other anticancer therapies [64]. A phase 1 trial of HCQ and temsirolimus (TEM) demonstrated that TEM and HCQ have antitumor effects by inhibiting autophagy in patient with a safe profile [65]. Another clinical study showed that the combination of high-dose HCQ and dose-intense temozolomide is safe and tolerable. The treatment outcome with a prolonged stable disease and responses suggested antitumor activity in melanoma patients [17]. These two clinical studies warranted the combination of potent autophagy modulating molecules for improving melanoma patients’ outcomes.

In melanoma, it has been shown that amodiaquine (AQ), a clinical 4-aminoquinoline antimalarial, can cause an autophagic–lysosomal and proliferative blockade in melanoma cells. It can also sensitize melanoma cells to either starvation- or chemotherapeutic agent-induced cell death [63]. Xie et al. also demonstrated that the antimalarial lysosomotropic agent HCQ synergized with TEM, which in turn led to the suppression of melanoma growth and induced cell death via apoptosis in both three-dimensional spheroid cultures and tumor xenografts [66]. In addition, McAfee et al. synthesized a bisaminoquinoline autophagy inhibitor Lys05, which is more potent than HCQ, and assessed its antitumor activity in vivo [67]. This result showed that mice bearing melanoma xenografts treated with Lys05 exhibited more significant tumor growth reduction than mice treated with PBS and HCQ [67]. The authors also demonstrated that Lys05 caused lysosomal dysfunction by deacidifying the lysosome, leading to the impairment of lysosomal enzymes and effective autophagy inhibition [67].

### 4.2. v-ATPase Inhibits Melanoma Survival and Metastasis

v-ATPase inhibition is another potential strategy for cancer therapeutics because cancer cells are more reliant on v-ATPase for survival than nontransformed cells [68]. In melanoma, Pan et al. demonstrated that cleistanthin A (CA), a natural compound which has the ability to inhibit v-ATPase activity and to neutralize the pH of lysosomes, inhibited the invasion and migration of human melanoma A375 cells in vitro by downregulating the expression of matrix metallopeptidase (MMP)-2 and -9 [69]. In line with this, another in vitro study also showed that concanamycin, a very specific inhibitor of v-ATPases, significantly suppressed the migration and invasion of murine melanoma B16F10 cells, but not nontumor melanocyte Melan-A cells [70]. Additionally, Martins et al. reported that the inhibition of v-ATPases by Myrtenal disrupted the electrochemical H+ gradient across the tumor cell membranes, leading to reprogrammed cell death and decreased tumor cell migration and invasion in vitro using murine as well as human melanoma cell lines [71]. Myrtenal also significantly suppressed metastasis induced by B16F10 murine melanoma in vivo, supporting v-ATPase as a molecular target to inhibit cancer progression [71]. Furthermore, the inhibition of v-ATPase by esomeprazole, a proton pump inhibitor, inhibited proliferation of human melanoma cells in vitro and reduced the tumor growth in vivo as well as increased survival of mice bearing human melanoma [72]. Moreover, Nishisho et al. identified a particular v-ATPase subunit, a3 isoform, as a potential therapeutic target by showing that knockdown of a3 v-ATPase in B16F10 murine melanoma model significantly reduced invasiveness and metastases to lung and bone [73].

### 4.3. Cathepsins Inhibition Deactivates Melanoma Invasiveness and Metastasis

Cathepsins have been correlated with cancer metastasis by facilitating cell migration and invasiveness due to their proteolytic activity. Hence, targeting cathepsins has potential to be a therapeutic strategy [74,75,76]. It is reported that inhibition of cathepsin B by either a specific chemical inhibitor, CA-074, or specific anti-cathepsin B antibodies significantly prevented the in vitro invasiveness of metastatic melanoma cell lines [76]. Accordingly, CA-074 dramatically reduced human melanoma growth and lung metastases in murine xenograft models [76]. Similarly, Liu et al. demonstrated that cepharanthine (CEP), a natural alkaloid with cathepsin B inhibitory function, inhibited human primary cutaneous melanoma cell viability and proliferation in vitro. It also decreased the tumor growth in cutaneous melanoma mouse models by the topical application or intra-tumoral injection of CEP [77]. In addition, the incubation of primary cutaneous melanoma cells with CEP showed not only decreased cathepsin B expression, but also decreased autophagy-related protein, LC3-I and LC3-II, expression in a dose-dependent manner, while antioncogene p53, p21Cip1p, and p16Inka expression was upregulated [77].

## 5. Conclusions

**Figure 1:**
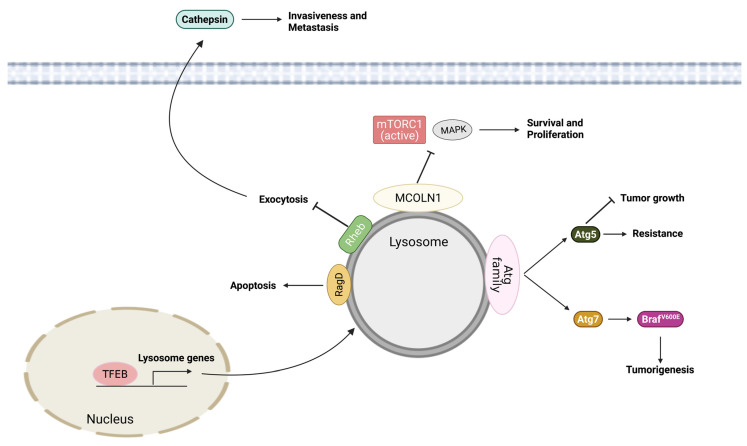
Scheme of the roles of lysosomes in melanoma.

The lysosome has emerged as a key regulatory hub in physiological homeostasis. Increasing evidence has also demonstrated its critical role in disease pathogenesis. Lysosomes are involved in many intracellular processes, including autophagy, and they have been shown to have a dual role in cancer progression and drug resistance. Therefore, to develop effective cancer therapeutics by targeting lysosomes, lysosomal activation and inhibition should be investigated cautiously. In addition, many lysosomotropic agents showing promising results are FDA approved and can translate to the clinic. A combination of lysosomotropic compounds with other therapies might improve the clinical outcomes of melanoma patients. However, more studies are needed to identify further lysosomotropic agents’ specific roles in lysosome biogenesis and metabolism. Similarly, it is important to determine the consequences of v-ATPase and cathepsin inhibition, although a variety of studies have demonstrated their potential as therapeutic targets. The purpose of this mini-review is to highlight the role of lysosomes in melanoma progression and therapy resistance and to encourage the development of lysosome-targeted therapeutics, which hold hope for improving clinical outcomes of melanoma patients (Figure 1).

## Figures and Tables

**Table 1:** Role of molecules related to lysosomal pathways in melanoma progression.

Molecule	Description	Role in Melanoma	Model	References
MCOLN1	MCOLN1 negatively regulates MAPK and mTORC1 signaling and sustains upregulated micropinocytosis	Survival and proliferation	Patient-derived melanoma	[19]
Atg7	Atg7 promotes the growth of BRAF^V600E^ melanoma	Melanomagenesis	Genetically engineered mouse models	[16]
Atg5	Downregulation of Atg5 promotes the proliferation of melanocytes introduced with mutated BRAF	Suppressing melanoma tumorigenesis	Primary melanocytes	[43]
LAMP-2C	Mice bearing melanoma cells with increased LAMP-2C expression show reduced tumor growth	Tumor suppressor in melanoma progression	Human melanoma cell lines (DM331 and SLM2-Mel)	[30]
Cathepsins B and L	Abl and Arg induce the secretion of cathepsins B and L; Cathepsin B and L inhibitors suppress the invasive growth of the melanoma cells	Metastasis and invasiveness	Human melanoma cell lines (WM115, WM793, WM239, SK-MEL-28, SK-MEL-103, SK-MEL-147, 435s, WM3248, and UACC-903)	[37,40]
Cathepsin K	Cathepsin K inhibition reduces melanoma cell invasion and increases detection of internalized collagen	Invasiveness	Human melanoma cell lines (MMAN, MeWo, and LIBR)	[38]
Rab7	Knockdown of Rab7 promotes the secretion of cathepsins and matrix proteins and subsequently increased melanoma invasion	Invasiveness	Human melanoma cell lines (SK-Mel-5, SK-Mel-19, SK-Mel-28, SK-Mel-29, SK-Mel-103, SK- Mel-147, SK-Mel-173, G-361, UACC-62, Mel-1, WM-164, 1205Lu and WM-1366)	[39]
MITF	Overexpression of MITF causes an expansion of the late endolysosome/MVB compartment and elevates expression of late endosomal proteins	Proliferation	Human melanoma cell lines (C32, 501Mel, SkMel28, and Lu1205); primary normal human epidermal melanocytes (NHEM)	[22,25,28]
RagD	RagD silencing causes reduced tumor growth in mice bearing MITF-dependent melanoma cells	Tumor growth	Human melanoma cell line (501Mel)	[29]
PRL3	PRL3 expression is inversely correlated with endolysosomal vesicle gene expression, and high PRL3 expression is associated with melanoma-specific death	Poor patient outcomes	Human melanoma cell lines (A375 and C092)	[41]

## Data Availability

Not applicable.

## Abbreviations

AQ	amodiaquine
Arg	Arginase
Atg5	Autophagy Related 5
Axin1	axis inhibition protein 1
bHLH-ZIP	basic helix-loop-helix leucine zipper
CA	cleistanthin A
CA-074	cathepsin B inhibitor
CEP	cepharanthine
Chk1	Checkpoint kinase 1
CLEAR	coordinated lysosomal expression and regulation
CYT	cytolytic activity
DDX21	DExD-Box Helicase 21
ER	Endoplasmic Reticulum
Ets1	ETS Proto-Oncogene 1, Transcription Factor
GSK3	Glycogen synthase kinase-3
HCQ	Hydroxychloroquine
LAMP	Lysosomal Associated Membrane Protein
LC3	light chain 3
Lys05	autophagy inhibitor
MCOLN1	Ca2+ channel mucolipin 1
MAPK	Mitogen-Activated Protein Kinase
MITF	micropthalmia-associated transcription factor
MiT/TFE	microphthalmia/transcription factor E
MMP	matrix metallopeptidase
mTORC1	mammalian target of rapamycin complex 1
MVB	multivesicular body
NF-*κ*B	nuclear factor kappa-light-chain-enhancer of activated B cells
NNMT	nicotinamide N-methyltransferase
p21	cyclin-dependent kinase inhibitor 1
PON2	paraoxonase-2
PRL3	Phosphatase of regenerating liver 3
Rab	Ras-Associated Protein
RagD	Ras related GTP binding D
ROS	reactive oxidative species
shRNA	short hairpin RNA
SNAP-23	Synaptosome Associated Protein 23
Sp1	Sp1 Transcription Factor
TEM	temsirolimus
TFE3	Transcription Factor Binding To IGHM Enhancer 3\
TFEB	Transcription Factor EB
TFEC	Transcription Factor EC
TGF-β	Transforming growth factor β
v-ATPase	Vacuolar-type ATPase
Wnt	Wingless and Int-1

## References

[1] Kallunki T., Olsen O.D., Jäättelä M. (2013). Cancer-associated lysosomal changes: Friends or foes?. Oncogene.

[2] Saftig P., Klumperman J. (2009). Lysosome biogenesis and lysosomal membrane proteins: Trafficking meets function. Nat. Rev. Mol. Cell Biol..

[3] Bonam S.R., Wang F., Muller S. (2019). Lysosomes as a therapeutic target. Nat. Rev. Drug Discov..

[4] Laikova K.V., Oberemok V.V., Krasnodubets A.M., Gal’Chinsky N.V., Useinov R.Z., Novikov I.A., Temirova Z.Z., Gorlov M.V., Shved N.A., Kumeiko V.V. (2019). Advances in the Understanding of Skin Cancer: Ultraviolet Radiation, Mutations, and Antisense Oligonucleotides as Anticancer Drugs. Molecules.

[5] Siegel R.L., Miller K.D., Fuchs H.E., Jemal A. (2022). Cancer statistics, 2022. CA A Cancer J. Clin..

[6] Coricovac D., Dehelean C., Moaca E.-A., Pinzaru I., Bratu T., Navolan D., Boruga O. (2018). Cutaneous Melanoma—A Long Road from Experimental Models to Clinical Outcome: A Review. Int. J. Mol. Sci..

[7] Domingues B., Lopes J.M., Soares P., Populo H. (2018). Melanoma treatment in review. Immuno Targets Ther..

[8] Zhang H., Liu Y., Hu D., Liu S. (2022). Identification of Novel Molecular Therapeutic Targets and Their Potential Prognostic Biomarkers Based on Cytolytic Activity in Skin Cutaneous Melanoma. Front. Oncol..

[9] Bacchetti T., Salvolini E., Pompei V., Campagna R., Molinelli E., Brisigotti V., Togni L., Lucarini G., Sartini D., Campanati A. (2020). Paraoxonase-2: A potential biomarker for skin cancer aggressiveness. Eur. J. Clin. Investig..

[10] Campagna R., Salvolini E., Pompei V., Pozzi V., Salvucci A., Molinelli E., Brisigotti V., Sartini D., Campanati A., Offidani A. (2021). Nicotinamide N-methyltransferase gene silencing enhances chemosensitivity of melanoma cell lines. Pigment Cell Melanoma Res..

[11] Hanahan D., Weinberg R.A. (2011). Hallmarks of cancer: The next generation. Cell.

[12] Lunt S.Y., Vander Heiden M.G. (2011). Aerobic Glycolysis: Meeting the Metabolic Requirements of Cell Proliferation. Annu. Rev. Cell Dev. Biol..

[13] Perera R.M., Zoncu R. (2016). The Lysosome as a Regulatory Hub. Annu. Rev. Cell Dev. Biol..

[14] Pizzimenti S., Ribero S., Cucci M.A., Grattarola M., Monge C., Dianzani C., Barrera G., Muzio G. (2021). Oxidative Stress-Related Mechanisms in Melanoma and in the Acquired Resistance to Targeted Therapies. Antioxidants.

[15] Catalani E., Giovarelli M., Zecchini S., Perrotta C., Cervia D. (2021). Oxidative Stress and Autophagy as Key Targets in Melanoma Cell Fate. Cancers.

[16] Xie X., Koh J.Y., Price S., White E., Mehnert J.M. (2015). *Atg7* Overcomes Senescence and Promotes Growth of *Braf*^V600E^-Driven Melanoma. Cancer Discov..

[17] Lawrence R.E., Zoncu R. (2019). The lysosome as a cellular centre for signalling, metabolism and quality control. Nat. Cell Biol..

[18] Tang T., Yang Z.-Y., Wang D., Yang X.-Y., Wang J., Li L., Wen Q., Gao L., Bian X.-W., Yu S.-C. (2020). The role of lysosomes in cancer development and progression. Cell Biosci..

[19] Kasitinon S.Y., Eskiocak U., Martin M., Bezwada D., Khivansara V., Tasdogan A., Zhao Z., Mathews T., Aurora A.B., Morrison S.J. (2019). TRPML1 Promotes Protein Homeostasis in Melanoma Cells by Negatively Regulating MAPK and mTORC1 Signaling. Cell Rep..

[20] Perera R.M., Di Malta C., Ballabio A. (2019). MiT/TFE Family of Transcription Factors, Lysosomes, and Cancer. Annu. Rev. Cancer Biol..

[21] Garraway L.A., Widlund H., Rubin M., Getz G., Berger A.J., Ramaswamy S., Beroukhim R., Milner J.D.A., Granter S.R., Du J. (2005). Integrative genomic analyses identify MITF as a lineage survival oncogene amplified in malignant melanoma. Nature.

[22] Ploper D., De Robertis E. (2015). The MITF family of transcription factors: Role in endolysosomal biogenesis, Wnt signaling, and oncogenesis. Pharmacol. Res..

[23] Tsao H., Chin L., Garraway L.A., Fisher D.E. (2012). Melanoma: From mutations to medicine. Genes Dev..

[24] Yokoyama S., Woods S.L., Boyle G.M., Aoude L.G., MacGregor S., Zismann V., Gartside M., Cust A.E., Haq R., Harland M. (2011). A novel recurrent mutation in MITF predisposes to familial and sporadic melanoma. Nature.

[25] Ploper D., Taelman V.F., Robert L., Perez B.S., Titz B., Chen H.-W., Graeber T.G., von Euw E., Ribas A., De Robertis E.M. (2015). MITF drives endolysosomal biogenesis and potentiates Wnt signaling in melanoma cells. Proc. Natl. Acad. Sci. USA.

[26] Bouché V., Espinosa A.P., Leone L., Sardiello M., Ballabio A., Botas J. (2016). *Drosophila* Mitf regulates the V-ATPase and the lysosomal-autophagic pathway. Autophagy.

[27] Zhang T., Zhou Q., Ogmundsdottir M.H., Möller K., Siddaway R., Larue L., Hsing M., Kong S.W., Goding C., Palsson A. (2015). Mitf is a master regulator of the v-ATPase forming an Mitf/v-ATPase/TORC1 control module for cellular homeostasis with v-ATPase and TORC1. J. Cell Sci..

[28] Möller K., Sigurbjornsdottir S., Arnthorsson A.O., Pogenberg V., Dilshat R., Fock V., Brynjolfsdottir S.H., Bindesboll C., Bessadottir M., Ogmundsdottir H.M. (2019). MITF has a central role in regulating starvation-induced autophagy in melanoma. Sci. Rep..

[29] Di Malta C., Siciliano D., Calcagni A., Monfregola J., Punzi S., Pastore N., Eastes A.N., Davis O., De Cegli R., Zampelli A. (2017). Transcriptional activation of RagD GTPase controls mTORC1 and promotes cancer growth. Science.

[30] Pérez L., Sinn A.L., Sandusky G.E., Pollok K.E., Blum J.S. (2018). Melanoma LAMP-2C Modulates Tumor Growth and Autophagy. Front. Cell Dev. Biol..

[31] Olson O.C., Joyce J.A. (2015). Cysteine cathepsin proteases: Regulators of cancer progression and therapeutic response. Nat. Rev. Cancer.

[32] Pan L., Li Y., Jia L., Qin Y., Qi G., Cheng J., Qi Y., Li H., Du J. (2012). Cathepsin S Deficiency Results in Abnormal Accumulation of Autophagosomes in Macrophages and Enhances Ang II–Induced Cardiac Inflammation. Pasterkamp, G., Ed. PLoS ONE.

[33] Dennemärker J., Lohmüller T., Müller S., Aguilar S.V., Tobin D.J., Peters C., Reinheckel T. (2010). Impaired turnover of autophagolysosomes in cathepsin L deficiency. Biol. Chem..

[34] Guinec N., Dalet-Fumeron V., Pagano M. (1993). “In vitro” Study of Basement Membrane Degradation by the Cysteine Proteinases, Cathepsins, B., B-Like and L. Digestion of Collagen IV, Laminin, Fibronectin, and Release of Gelatinase Activities front Basement Membrane Fibronectin. Biol. Chem. Hoppe-Seyler.

[35] Gocheva V., Wang H.-W., Gadea B.B., Shree T., Hunter K.E., Garfall A.L., Berman T., Joyce J.A. (2010). IL-4 induces cathepsin protease activity in tumor-associated macrophages to promote cancer growth and invasion. Genes Dev..

[36] Sevenich L., Bowman R.L., Mason S.D., Quail D.F., Rapaport F., Elie B.T., Brogi E., Brastianos P.K., Hahn W.C., Holsinger L.J. (2014). Analysis of tumour- and stroma-supplied proteolytic networks reveals a brain-metastasis-promoting role for cathepsin S. Nat. Cell Biol..

[37] Yin M., Soikkeli J., Jahkola T., Virolainen S., Saksela O., Hölttä E. (2012). TGF-β Signaling, Activated Stromal Fibroblasts, and Cysteine Cathepsins B and L Drive the Invasive Growth of Human Melanoma Cells. Am. J. Pathol..

[38] Quintanilla-Dieck M.J., Codriansky K., Keady M., Bhawan J., Rünger T.M. (2008). Cathepsin K in Melanoma Invasion. J. Investig. Dermatol..

[39] Curbelo D.A., Riveiro-Falkenbach E., Pérez-Guijarro E., Cifdaloz M., Karras P., Osterloh L., Megias D., Cañón E., Calvo T.G., Olmeda D. (2014). RAB7 Controls Melanoma Progression by Exploiting a Lineage-Specific Wiring of the Endolysosomal Pathway. Cancer Cell.

[40] Tripathi R., Fiore L.S., Richards D.L., Yang Y., Liu J., Wang C., Plattner R. (2018). Abl and Arg mediate cysteine cathepsin secretion to facilitate melanoma invasion and metastasis. Sci. Signal..

[41] Johansson J.A., Marie K.L., Lu Y., Brombin A., Santoriello C., Zeng Z., Zich J., Gautier P., von Kriegsheim A., Brunsdon H. (2020). PRL3-DDX21 Transcriptional Control of Endolysosomal Genes Restricts Melanocyte Stem Cell Differentiation. Dev. Cell.

[42] Michalowski (2019). Melanoblast Transcriptome Analysis Reveals Novel Pathways Promoting Melanoma Metastasis. Marie et al..

[43] Liu H., He Z., Simon H.-U. (2013). Autophagy suppresses melanoma tumorigenesis by inducing senescence. Autophagy.

[44] Soengas M.S., Lowe S.W. (2003). Apoptosis and melanoma chemoresistance. Oncogene.

[45] Wilson M.A., Schuchter L.M., Kaufman H.L., Mehnert J.M. (2016). Chemotherapy for Melanoma. Melanoma.

[46] Kalal B.S., Upadhya D., Pai V.R. (2017). Chemotherapy resistance mechanisms in advanced skin cancer. Oncol. Rev..

[47] Zhitomirsky B., Assaraf Y.G. (2016). Lysosomes as mediators of drug resistance in cancer. Drug Resist. Updat..

[48] Sui X., Chen R., Wang Z., Huang Z., Kong N., Zhang M., Han W., Lou F., Yang J., Zhang Q. (2013). Autophagy and chemotherapy resistance: A promising therapeutic target for cancer treatment. Cell Death Dis..

[49] Ma X.-H., Piao S., Wang D., McAfee Q.W., Nathanson K., Lum J.J., Li L., Amaravadi R.K. (2011). Measurements of Tumor Cell Autophagy Predict Invasiveness, Resistance to Chemotherapy, and Survival in Melanoma. Clin. Cancer Res..

[50] Li S., Song Y., Quach C., Guo H., Jang G.-B., Maazi H., Zhao S., Sands N.A., Liu Q., In G.K. (2019). Transcriptional regulation of autophagy-lysosomal function in BRAF-driven melanoma progression and chemoresistance. Nat. Commun..

[51] Tanda E.T., Vanni I., Boutros A., Andreotti V., Bruno W., Ghiorzo P., Spagnolo F. (2020). Current State of Target Treatment in BRAF Mutated Melanoma. Front. Mol. Biosci..

[52] Ma X.-H., Piao S.-F., Dey S., McAfee Q., Karakousis G., Villanueva J., Hart L.S., Levi S., Hu J., Zhang G. (2014). Targeting ER stress–induced autophagy overcomes BRAF inhibitor resistance in melanoma. J. Clin. Investig..

[53] García-Fernández M., Karras P., Checinska A., Cañón E., Calvo G.T., López G.G., Cifdaloz M., Colmenar A., Espinosa-Hevia L., Olmeda D. (2016). Metastatic risk and resistance to BRAF inhibitors in melanoma defined by selective allelic loss of ATG5. Autophagy.

[54] Law R.H.P., Lukoyanova N., Voskoboinik I., Caradoc-Davies T.T., Baran K., Dunstone M., D’Angelo M., Orlova E., Coulibaly F., Verschoor S. (2010). The structural basis for membrane binding and pore formation by lymphocyte perforin. Nature.

[55] Khazen R., Müller S., Gaudenzio N., Espinosa E., Puissegur M.-P., Valitutti S. (2016). Melanoma cell lysosome secretory burst neutralizes the CTL-mediated cytotoxicity at the lytic synapse. Nat. Commun..

[56] Santana-Magal N., Farhat-Younis L., Gutwillig A., Gleiberman A., Rasoulouniriana D., Tal L., Netanely D., Shamir R., Blau R., Feinmesser M. (2020). Melanoma-Secreted Lysosomes Trigger Monocyte-Derived Dendritic Cell Apoptosis and Limit Cancer Immunotherapy. Cancer Res..

[57] Towers C.G., Thorburn A. (2017). Targeting the Lysosome for Cancer Therapy. Cancer Discov..

[58] Xie J., Wang X., Proud C.G. (2016). mTOR inhibitors in cancer therapy. F1000Research.

[59] Levy J.M.M., Towers C.G., Thorburn A. (2017). Targeting autophagy in cancer. Nat. Cancer.

[60] Geisslinger F., Müller M., Vollmar A.M., Bartel K. (2020). Targeting Lysosomes in Cancer as Promising Strategy to Overcome Chemoresistance—A Mini Review. Front. Oncol..

[61] Aits S., Jäättelä M. (2013). Lysosomal cell death at a glance. J. Cell Sci..

[62] Weir S.J., DeGennaro L.J., Austin C.P. (2012). Repurposing Approved and Abandoned Drugs for the Treatment and Prevention of Cancer through Public–Private Partnership: Figure 1. Cancer Res..

[63] Qiao S., Tao S., De La Vega M.R., Park S.L., Vonderfecht A.A., Jacobs S.L., Zhang D.D., Wondrak G.T. (2013). The antimalarial amodiaquine causes autophagic-lysosomal and proliferative blockade sensitizing human melanoma cells to starvation- and chemotherapy-induced cell death. Autophagy.

[64] Piao S., Amaravadi R.K. (2015). Targeting the lysosome in cancer: Targeting the lysosome in cancer. Ann. N. Y. Acad. Sci..

[65] Rangwala R., Chang Y.C., Hu J., Algazy K.M., Evans T.L., Fecher L.A., Schuchter L.M., Torigian D.A., Panosian J.T., Troxel A.B. (2014). Combined MTOR and autophagy inhibition. Phase I trial of hydroxychloroquine and temsirolimus in patients with advanced solid tumors and melanoma. Autophagy.

[66] Xie X., White E.P., Mehnert J.M. (2013). Coordinate Autophagy and mTOR Pathway Inhibition Enhances Cell Death in Melanoma. PLoS ONE.

[67] McAfee Q., Zhang Z., Samanta A., Levi S.M., Ma X.-H., Piao S., Lynch J.P., Uehara T., Sepulveda A.R., Davis L.E. (2012). Autophagy inhibitor Lys05 has single-agent antitumor activity and reproduces the phenotype of a genetic autophagy deficiency. Proc. Natl. Acad. Sci. USA.

[68] Stransky L., Cotter K., Forgac M. (2016). The Function of V-ATPases in Cancer. Physiol. Rev..

[69] Pan S., Cai H., Gu L., Cao S. (2017). Cleistanthin A inhibits the invasion and metastasis of human melanoma cells by inhibiting the expression of matrix metallopeptidase-2 and -9. Oncol. Lett..

[70] Costa G.A., de Souza S.B., Teixeira L.R.D.S., Okorokov L.A., Arnholdt A.C.V., Okorokova-Façanha A., Façanha A.R. (2018). Tumor cell cholesterol depletion and V-ATPase inhibition as an inhibitory mechanism to prevent cell migration and invasiveness in melanoma. Biochim. Biophys. Acta BBA Gen. Subj..

[71] Martins B.X., Arruda R.F., Costa G.A., Jerdy H., de Souza S.B., Santos J.M., de Freitas W.R., Kanashiro M.M., de Carvalho E.C.Q., Sant’Anna N.F. (2018). Myrtenal-induced V-ATPase inhibition—A toxicity mechanism behind tumor cell death and suppressed migration and invasion in melanoma. Biochim. Biophys. Acta BBA Gen. Subj..

[72] De Milito A., Canese R., Marino M.L., Borghi M., Iero M., Villa A., Venturi G., Lozupone F., Iessi E., Logozzi M. (2009). pH-dependent antitumor activity of proton pump inhibitors against human melanoma is mediated by inhibition of tumor acidity. Int. J. Cancer.

[73] Nishisho T., Hata K., Nakanishi M., Morita Y., Sun-Wada G.-H., Wada Y., Yasui N., Yoneda T. (2011). The *a*3 Isoform Vacuolar Type H^+^-ATPase Promotes Distant Metastasis in the Mouse B16 Melanoma Cells. Mol. Cancer Res..

[74] Guicciardi M.E., Leist M., Gores G.J. (2004). Lysosomes in cell death. Oncogene.

[75] Podgorski I., Sloane B.F. (2003). Cathepsin B and its role(s) in cancer progression. Biochem. Soc. Symp..

[76] Matarrese P., Ascione B., Ciarlo L., Vona R., Leonetti C., Scarsella M., Mileo A.M., Catricalà C., Paggi M.G., Malorni W. (2010). Cathepsin B inhibition interferes with metastatic potential of human melanoma: An in vitro and in vivo study. Mol. Cancer.

[77] Liu Y., Xie Y., Lin Y., Xu Q., Huang Y., Peng M., Lai W., Zheng Y. (2020). Cepharanthine as a Potential Novel Tumor-Regional Therapy in Treating Cutaneous Melanoma: Altering the Expression of Cathepsin B, Tumor Suppressor Genes and Autophagy-Related Proteins. Front. Bioeng. Biotechnol..

